# Ezetimibe reduced hepatic steatosis induced by dietary oxysterols in nonhuman primates

**DOI:** 10.1002/2211-5463.12107

**Published:** 2016-09-20

**Authors:** Michiyo Deushi, Mizuko Osaka, Kaku Nakano, Kyoichi Osada, Kensuke Egashira, Masayuki Yoshida

**Affiliations:** ^1^ Department of Life Sciences and BioethicsGraduate School of Medical and Dental SciencesTokyo Medical and Dental UniversityJapan; ^2^ Department of Cardiovascular MedicineGraduate School of Medical SciencesKyushu UniversityFukuokaJapan; ^3^ Department of Agricultural ChemistrySchool of AgricultureMeiji UniversityKanagawaJapan

**Keywords:** dietary oxysterols, ezetimibe, Niemann–Pick C1‐like1, nonalcoholic fatty liver disease, nonhuman primate, steatosis

## Abstract

Oxidized cholesterol (oxysterols) plays an important and multifaceted role in lipid metabolism. Here we examined whether dietary oxysterols accelerate hepatic lipid accumulation and inflammation in nonhuman primates. We also examined the effect of the Niemann–Pick C1‐like1 inhibitor, ezetimibe (Ez). *Macaca fascicularis* (5‐year‐old males) were fed either regular cholesterol + high‐fat diet (control‐HFD) or oxysterols + high‐fat diet (ox‐HFD; with 0.015% of oxysterols cholesterol) for 24 weeks. Compared with control‐HFD, ox‐HFD did not affect plasma lipid levels, but it did affect hepatic lipid levels [total cholesterol, 40.9 mg·g^−1^ (ox‐HFD) versus 3.2 (control‐HFD) mg·g^−1^; triglycerides, 28.0 (ox‐HFD) versus 5.7 (control‐HFD) mg·g^−1^]. Ox‐HFD increased lipid accumulation as well as recruitment of inflammatory cells when compared to control‐HFD. We then examined the effects of Ez, 0.2 mg·kg^−1^·day^−1^ for 12 weeks. In addition to a significant reduction in dyslipidemia, Ez alleviated biochemical and pathological aspects of steatosis. Dietary oxysterols aggravate steatosis in nonhuman primates. Treatment with Ez may be a novel therapeutic approach to NAFLD by alleviating dyslipidemia.

**Abbreviations**
ALT: alanine aminotransferase
AST: aspartate aminotransferase
CE: cholesterylester
CMC‐Na: carboxymethyl cellulose
DMSO: dimethyl sulfoxide
Ez: ezetimibe
FC: free cholesterol
HDL: high‐density lipoprotein
HFD: high‐fat diet
HMGcoA: hydroxymethylglutaryl‐coenzyme A
HPLC: high‐performance liquid chromatography
IFN: interferon
IL: interleukin
LDL: low‐density lipoprotein
LXR: liver X receptor
NAFLD: nonalcoholic fatty liver disease
NASH: nonalcoholic steatohepatitis
NKT: natural killer T
NPC1L1: Niemann–Pick C1‐like1
SCF: stem cell factor
TC: total cholesterol
TG: triacylglycerol
VEGF: vascular endothelial growth factor
VLDL: very low‐density lipoprotein

Dietary cholesterol is easily oxidized by exposure to oxygen (prolonged storage) or high temperatures (cooking or reheating). Cholesterol in foods is readily oxidized in animal food products and fast foods 1, 2. It is critically important to determine the effect on human health of oxidized cholesterol (oxysterols) in the diet with today's lifestyle modifications. Previous studies suggest proinflammatory effects of meals rich in cholesterol, particularly oxysterols 3. In addition, oxysterols in food has an important role in accelerating atherosclerosis 4.

Nonalcoholic fatty liver disease (NAFLD) manifests with lipid accumulation in hepatocytes, followed by inflammation and fibrosis. Recent clinical studies suggest that NAFLD is a major comorbid condition in obesity and other metabolic disorders, including diabetes. Dietary fat intake elevates serum cholesterol and triglyceride (TG) levels, and excessive fat accumulates in the adipose tissue and liver. This initial accumulation of lipid in the liver (steatosis) then becomes more pathogenic inflammation. Notably, 30% of steatosis patients were diagnosed with steatohepatitis 5. Thus, fatty liver should be considered as an early stage of metabolic syndrome in the liver. Although previous studies claim the importance of dietary fat intake in NAFLD, its qualitative effect has been not exclusively studied.

Moreover, in previous reports, free cholesterol directly affects hepatic mitochondrial glutathione degradation which leads to steatohepatitis 6.

There are some reports that dietary oxysterols may induce NAFLD or nonalcoholic steatohepatitis 7 in experimental animals. However, there is no established theory and the manner in which dietary oxysterols affect lipid metabolism in the liver or whole body is controversial. Differences in lipid metabolism in rodents make it difficult to apply these data to humans 8. Thus, for the present study, we utilized nonhuman primates as subjects as their lipid metabolism is similar to ours. Here we demonstrate that oxysterols intake significantly accelerates the development of NAFLD in nonhuman primates.

Ezetimibe (Ez), a potent inhibitor of cholesterol absorption, reverses hypercholesterolemia 9. Our group recently reported its effect on NAFLD in a rat model of the metabolic syndrome. We could document a role of hepatic Niemann–Pick C1‐like1 (NPC1L1), a target of Ez, in the development of hepatic inflammation induced by cholesterol accumulation 10. However, in rodents, low expression levels of NPC1L1 in the liver limited our study. Because NPC1L1 expression in liver is as high in nonhuman primates as that in the human intestine, we decided to study the role of oxidative cholesterol products in nonhuman primates.

## Materials and methods

### Reagents

Ez was supplied by MSD, Tokyo, Japan. Ez was resolved in dimethyl sulfoxide (DMSO; Wako Pure Chemical Industry Ltd., Tokyo, Japan), and DMSO was used as a control. Antibodies used in this study were as follows: anti‐smooth muscle actin (Sigma, St. Louis, MO, USA), anti‐CD11b (Abcam, Cambridge, UK), and anti‐CD68 (KP1, Santa Cruz, CA, USA).

### Preparation of oxysterols

Oxysterols for oxysterols + high‐fat diet (ox‐HFD) was prepared from pure cholesterol (Riken Vitamin, Tokyo, Japan) by heating, as previously described 11. In brief, the cholesterol was heated in an electric oven at ~ 150 °C for 12 h. Heated cholesterol was dissolved in n‐hexane, applied to a silicic acid column (Silicagel 60, 70–230 mesh; 100 mm × 800 mm) (E. Merck, Darmstadt, Germany) and fractionated by successive elution with 1 L each of n‐hexane, diethyl ether, and methanol. An aliquot of the polar fraction eluted by methanol was dried in a rotary evaporator, and then, *in vacuo*. The compositions are described in Table 1.

**Table 1:** Composition of oxysterols made from control cholesterol, as described in the Materials and methods section

Oxysterols	% (wt/wt)
7α‐Hydroxycholesterol	3.7
7β‐Hydroxycholesterol	4.3
5α‐Epoxycholesterol	0.5
5β‐Epoxycholesterol	3.9
Cholestane‐3β, 5α, 6β‐triol	1.6
7‐Ketocholesterol	4.4
Unidentified oxysterols	13.0
Total oxysterols	31.4
Total cholesterol	68.0

### Animals and experimental design

All animal experiments were conducted by Animal Experimentation of Primates Inc. (Hangzhou, China). All experimental procedures conformed to the APS Guiding Principles in the Care and Use Animals and received the approval of the Ethical Committee of Animal Experimentation of Primates Inc. Cynomolgus monkeys (*Macaca fascicularus*; 5‐year‐old males) were fed 150 g·day^−1^ of a regular HFD (21% lard, with 2% cholesterol). In addition, HFD with 2.1 g of control cholesterol (control‐HFD) or 0.015% oxysterols (ox‐HFD) was dissolved in 0.5% carboxymethyl cellulose (CMC‐Na; Wako) and force‐fed orally once per day for 24 weeks. Water was provided *ad libitum*. In some animals, Ez (0.2 mg·kg^−1^·day^−1^) dissolved in 0.5% CMC‐Na was orally administered once per day for the final 12 weeks (ox‐HFD + Ez).

### Lipoprotein analysis

Plasma lipoproteins were analyzed by the online dual enzymatic method for simultaneous quantification of cholesterol and TG by high‐performance liquid chromatography (HPLC) at Skylight Biotech (Akita, Japan), as described previously 12.

### Tissue morphology and immunohistochemistry

Liver samples were fixed in 10% buffered formalin for embedding in paraffin. For the Masson's trichrome staining, 5‐μm sections were deparaffinized, rehydrated, and stained with eosin as a counter stain. The antibodies were stained using an ABC‐AP kit and AEC kit (Vector Laboratories, Cambridgeshire, UK), according to standard procedures.

### Liver lipid analysis

Liver was rapidly removed postmortem. Lipids were extracted from liver tissue according to methods modified from that of Folch *et al*. 13. Briefly, snap‐frozen livers were homogenized after freeze‐drying and extracted with a chloroform/methanol (2 : 1 v/v) solution. The organic phase was dried by water bath (60 °C) and resolubilized in 2‐propanol containing 10% Triton X‐100. For TG, liver tissue was freeze‐dried and then extracted with 2‐propanol. Total cholesterol (TC) and TG levels were determined by enzymatic kits (Wako Pure Chemical Industry Ltd), as described previously 14, 15.

### Antibody array

Snap‐frozen livers from three animals exposed to each condition were pooled and homogenized in lysis buffer, as described previously 16. Lysates were analyzed according to the manufacturer's instructions, with a Human Cytokine Array C3 (RayBiotech, Norcross, GA, USA).

### Statistical analysis

Data are expressed as mean ± standard error of mean. One‐way analysis of variance with a Tukey *post hoc* test or two‐tailed unpaired *t*‐test was used to analyze statistical significance. A value of *P* < 0.05 was considered statistically significant.

## Results

### Ez improved dietary oxysterols‐induced hypercholesterolemia

**Figure 1:**
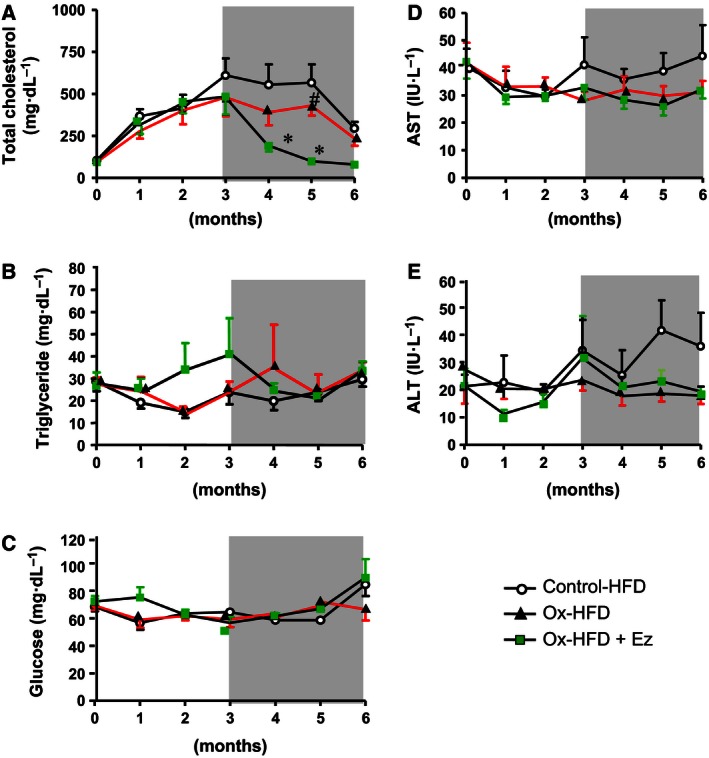
Plasma lipid profiles (*n* = 6). After the initial 3 months (bright area) of control‐HFD (open circle) and ox‐HFD (filled black triangle), the same conditions were either continued or ox‐HFD plus Ez treatment (filled square) was administered for the next 3 months (dark area). TC (A), TG (B), glucose (C; mg·dL^−1^), and hepatic enzyme [aspartate aminotransferase (D) and alanine aminotransferase (E); IU·L^−1^] levels were determined using enzymatic reagents. #*P* < 0.05, *P < 0.01

In the initial 3‐month period, plasma cholesterol levels were elevated in the ox‐HFD group as well as the control‐HFD group. After 1 month of Ez treatment (at the fourth month), plasma cholesterol levels significantly decreased in ox‐HFD‐treated animals than those in ox‐HFD‐untreated animals (average, 191.2 versus 389.0 mg·dL^−1^, *P* < 0.01; Fig. 1A); however, no significant differences were observed in plasma TG levels (Fig. 1B). Plasma glucose and hepatic enzyme levels (aspartate aminotransferase and alanine aminotransferase) did not significantly change throughout the study periods (Fig. 1C–E).

**Figure 2:**
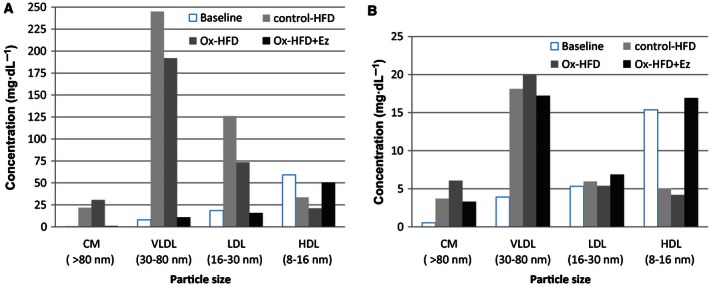
Lipoprotein profiles at the end point (pooled plasma). Cholesterol (A) and TG (B) content in chylomicron, VLDL, LDL, and HDL were determined using enzymatic reagents. Baseline levels (white) and those for control‐HFD (light gray), ox‐HFD (dark gray), and ox‐HFD plus Ez treatment (black) groups, as described in the Materials and methods section.

To monitor cholesterol and TG levels in lipoprotein particles, six plasma samples were pooled and subjected to HPLC analysis. Both cholesterol and TG in more atherogenic lipoproteins including chylomicrons, very low‐density lipoprotein (VLDL), and low‐density lipoprotein (LDL) particles showed a tendency to increase after control‐ or ox‐HFD treatments. Notably, after control‐HFD treatment, the plasma cholesterol levels were higher compared with those after ox‐HFD treatment (VLDL, 245.14 versus 191.91 mg·dL^−1^; LDL, 126.01 versus 73.50 mg·dL^−1^; Fig. 2A). These dyslipidemic conditions were strongly alleviated by Ez treatment [VLDL, 191.91 to 10.81 mg·dL^−1^; LDL, 73.50 to 15.79 mg·dL^−1^; high‐density lipoprotein (HDL), 21.19 to 50.49 mg·dL^−1^; Fig. 2A]. In contrast, HDL‐TG levels, which decreased in control‐HFD and ox‐HFD groups, slightly recovered after Ez treatment (Fig. 2B).

### Ezetimibe alleviated dietary oxysterols‐induced steatosis

**Figure 3:**
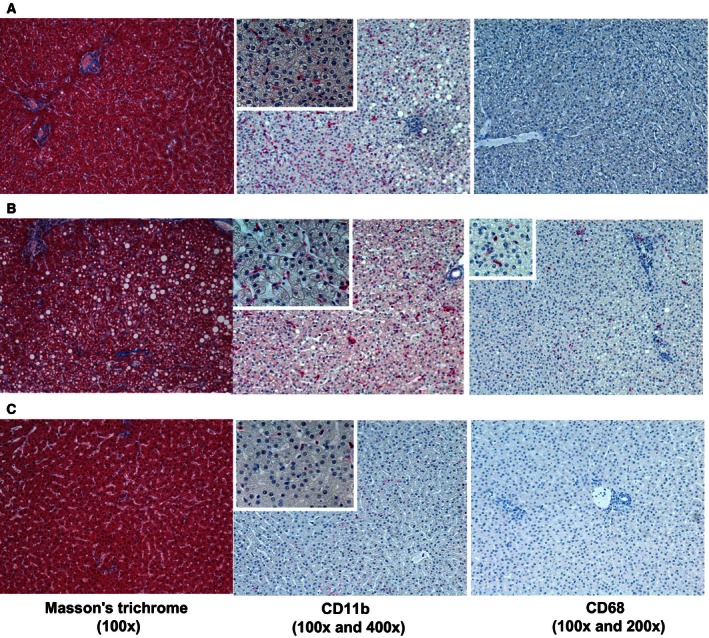
Liver histology. Masson's trichrome staining and immunohistochemistry (for CD11b and CD68). Control‐HFD (A), ox‐HFD (B), and ox‐HFD plus Ez treatment (C). Magnifications used 100× and 200× or 400× in small boxes.

**Figure 4:**
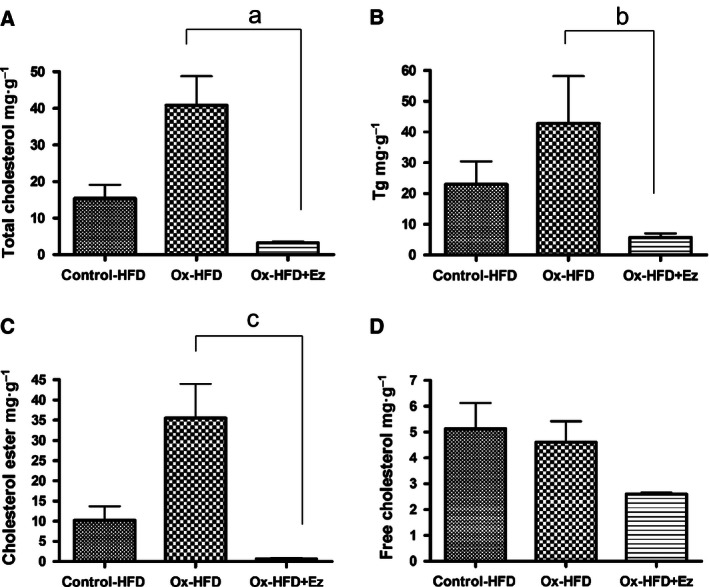
Hepatic lipids (*n* = 3). Cholesterol ester levels were calculated by multiplying each measurement. TC, total cholesterol; FC, free cholesterol; CE, cholesterylester; TG, triglyceride. Control‐HFD (solid bar), ox‐HFD (checked bar), and ox‐HFD plus Ez treatment (bordered bar). ^a^ *P* = 0.004, ^b^ *P* = 0.0984, ^c^ *P* = 0.0056.

We then examined the impact of oxysterols on liver steatosis and its potential modulation by Ez. As shown in Fig. 3, massive lipid accumulation and presence of CD11b‐ and CD68‐positive cells were observed in the ox‐HFD group when compared to the control‐HFD group. We also assessed the effect of Ez on ox‐HFD‐induced liver steatosis. Ez abolished the enhanced expression of CD11b and CD68 by oxysterols treatment, suggesting a favorable effect of Ez on HF‐induced liver fibrosis as well as steatosis (Fig. 3). Ez affected hepatic lipid content [TC 40.9 (ox‐HFD) versus 3.2 (control‐HFD) mg·g^−1^; TG, 28.0 (ox‐HFD) versus 5.7 (control‐HFD) mg·g^−1^; Fig. 4A,D]. TC level in the liver was decreased by Ez. Further analysis revealed that the level of cholesterylester (CE) but not free cholesterol (FC) was reduced by Ez (Fig. 4B,C).

### Adding dietary oxysterols and ezetimibe changed proinflammatory cytokine pattern of liver

As shown in Table 2, levels of cytokines including interleukin (IL)‐13, IL1‐α, IL1‐β, interferon‐γ, and angiotensin were increased after ox‐HFD intake; these were then decreased by Ez treatment. Conversely, ox‐HFD decreased the levels of cytokines including IL‐12p40p70, IL‐8, GRO, stem cell factor, and vascular endothelial growth factor; these were increased by Ez treatment (Table 2).

**Table 2:** Comparison of the expression of cytokines by antibody array (pooled liver homogenates). Expressed by a ratio of the control‐HFD as 1.00

	Ox‐HFD
IL13	4.50
IL1α	2.11
IL1β	1.77
IFNγ	1.25
Angiotensin	1.15
IGF1	1.04
control‐HFD = 1.00	
IL10	0.95
MCP1	0.94
IL2	0.87
VEGF	0.84
SCF	0.78
GRO	0.69
IL8	0.67
IL12p40p70	0.57

	Ox‐HFD + Ez
GRO	2.48
IL10	1.87
MCP1	1.77
IL2	1.27
VEGF	1.27
IL8	1.17
IL12p40p70	1.11
SCF	1.05
control‐HFD = 1.00	
IL1α	0.90
IFNγ	0.72
IL1β	0.64
Angiotensin	0.54
IGF1	0.52
IL13	0.00

## Discussion

NAFLD is a chronic liver disease, with manifestations ranging from simple lipid accumulation in hepatocytes (steatosis) with inflammation (steatohepatitis) to fibrosis (finally cirrhosis) in the absence of alcohol‐induced liver damage 17. It is characterized by macrovesicular lipid accumulation in hepatocytes with acute and chronic inflammation and perivenular deposition of extracellular matrix. An emerging role for lipotoxicity in the development of NAFLD has been recently reported.

As shown in Fig. 1, plasma TC levels were higher in control‐HFD group than in ox‐HFD group. This may be due to the inhibiting action of oxysterols on cholesterol absorption or hydroxymethyl glutaryl‐coenzyme A (HMGcoA) reductase 18, 19. In contrast, hepatic inflammation was aggravated in ox‐HFD group compared with that in control‐HFD group. Our immunohistochemical analysis revealed massive inflammation with infiltration of CD68‐positive macrophages in ox‐HFD group (Fig. 3), although we could not find a quantitative difference between control‐HFD versus ox‐HFD. Moreover, oxysterols upregulated IL‐13 expression in the liver. IL‐13 secreted from natural‐killer T (NKT) cells 20 has been shown to progress liver fibrosis 21 in NAFLD. NKT cells may play an important role in oxysterols‐mediated hepatic inflammation.

We also observed a potential effect of Ez on oxidative cholesterol‐induced NAFLD. As shown in Fig. 1, plasma TG and cholesterol levels were significantly reduced in the Ez‐treated ox‐HFD group compared with the untreated ox‐HFD group. Furthermore, Ez decreased hepatic TG and cholesterol levels (Fig. 4). This observation is consistent with previous studies 18, supporting and extending the notion that dietary oxysterols can be absorbed by the small intestine via NPC1L1 in nonhuman primates.

NPC1L1 is expressed in both the small intestine and liver in human and primates. However, the NPC1L1 that is important for the effects of oxysterols remains unknown. Sugizaki *et al*. 22 reported that Ez treatment may decrease liver X receptor (LXR) activity. Thus, an LXR‐mediated alternative pathway may influence the net intake of oxysterols independent of NPC1L1‐dependent absorption. Further studies will be required to elucidate the direct effects of oxysterols on enterocytes and hepatocytes. As we previously reported 10, the cholesterol absorption pathway varies among species. Thus, the current study using nonhuman primates is advantageous in the context of clinical relevancy.

Oxidative stress caused by oxysterols may play an important role in various pathogenic conditions, because abnormal oxidative stress has been observed in atherosclerosis and NAFLD 23, 24. Therefore, using a similar model, oxidative stress alone needs to be examined.

## Conclusion

We reported that Ez treatment rescued steatosis and changed the hepatic cytokine balance, conditions which were aggravated by dietary oxysterols in nonhuman primates. Further study will uncover the yet unknown mechanisms underpinning the relationship between dietary intake of oxysterols and NAFLD.

## Author contributions

MD collected the data and wrote the manuscript. MO, KO, and KN gave advice as specialists. KE and MY reviewed and edited the manuscript.
